# A Population-Based Cohort Study on the Association of Hyperthyroidism With the Risk of Hyperlipidemia and the Effects of Anti-thyroid Drugs on Hepatic Gene Expression

**DOI:** 10.3389/fmed.2020.00228

**Published:** 2020-05-29

**Authors:** Tien-Yuan Wu, Chung-Hsing Wang, Ni Tien, Cheng-Li Lin, Fang-Yi Chu, Hsiao-Yun Chang, Yun-Ping Lim

**Affiliations:** ^1^Department of Pharmacy, Taichung Tzu Chi Hospital, Buddhist Tzu Chi Medical Foundation, Taichung City, Taiwan; ^2^Department of Pharmacology, School of Medicine, Tzu Chi University, Hualien City, Taiwan; ^3^Children's Hospital of China Medical University, Taichung City, Taiwan; ^4^Department of Laboratory Medicine, China Medical University Hospital, Taichung City, Taiwan; ^5^Department of Medical Laboratory Science and Biotechnology, China Medical University, Taichung City, Taiwan; ^6^Management Office for Health Data, China Medical University Hospital, Taichung City, Taiwan; ^7^Department of Pharmacy, College of Pharmacy, China Medical University, Taichung City, Taiwan; ^8^Department of Biotechnology, Asia University, Taichung City, Taiwan; ^9^Department of Internal Medicine, China Medical University Hospital, Taichung City, Taiwan; ^10^Department of Medical Research, China Medical University Hospital, Taichung City, Taiwan

**Keywords:** hyperthyroidism, anti-thyroid drugs (ATDs), hyperlipidemia, Longitudinal Health Insurance Database (LHID), retrospective cohort study

## Abstract

There have been no reports on the association of hyperthyroidism with hyperlipidemia in patients undergoing treatment especially in Asia. To determine the association between hyperthyroidism and the risk of hyperlipidemia in patients, we conducted a retrospective cohort study using Longitudinal Health Insurance Database (LHID) from Taiwan, R.O.C. We also evaluate the influence of 6-*n*-propyl-2-thiouracil (PTU) and methimazole (MMI) on hepatic genes to explain changes in blood lipid levels in a hepatic cell line model. The cohort study involved 13,667 patients with hyperthyroidism, and the corresponding comparison cohort had four times as many patients. Using Kaplan-Meier analysis method, the results showed that, compared to patients without hyperthyroidism, the overall incidence of hyperlipidemia was significantly higher in the hyperthyroidism patients (18.7 vs. 11.8 cases/1,000 persons-years; adjusted HR 1.5; 95% CI, 1.41–1.59). With only PTU or MMI/carbimazole (CBM) treatment, patients with hyperthyroidism showed a 1.78-fold (95% CI, 1.50–2.11) and 1.43-fold (95% CI, 1.27–1.60) higher risk of hyperlipidemia than those without hyperthyroidism, respectively. Additionally, hyperthyroidism patients that received surgery only or surgery with I^131^ therapy tended to have a higher risk of hyperlipidemia. Although PTU and MMI treatment decreased the expression levels of genes responsible for circulating remnant lipoproteins, they increased the levels of lipogenic gene expression in hepatic cells. Thus, treatment of hyperthyroid patients with anti-thyroid drugs (ATDs), I^131^, or surgery is likely to induce hyperlipidemia. ATDs downregulate the expression of genes involved in lipoproteins clearance; increases lipogenic genes expression, which may partly contribute to abnormal blood lipid profiles.

## Introduction

Thyroid is an important organ involved in the regulation of various cellular processes such as cell proliferation and development, control of resting metabolic rate, thermoregulation, and metabolism of carbohydrates, proteins, and lipids (1). Dysfunction of thyroid affects appetite, body weight, muscle mass, as well as brings about changes in the adipose tissue by altering lipolysis, resulting in increased incidences of insulin resistance, type 2 diabetes mellitus (T2DM), and cardiovascular diseases (CVDs) (2). It has also been shown that patients with thyroid dysfunction have significantly altered lipids profiles.

Hypo- and hyperthyroidism are two of the most common diseases of the thyroid gland. Hyperthyroidism is a prevalent endocrine disorder, which is characterized by excessive secretion of the thyroid hormones, triiodothyronine (T_3_) and thyroxine (T_4_) (1). Graves' disease (GD) is an organ-specific autoimmune disorder that is caused by thyroid stimulatory immunoglobulins and it represents the most common type of hyperthyroidism (3). The autoantibodies produced imitate the thyroid stimulating hormone (TSH) and lead to stimulation of thyroid function and thus, suppress the TSH levels while elevating serum free T_4_ and T_3_ levels.

A decrease in the thyroid hormone levels is associated with an abnormal elevation of serum lipids and this increases the risk of CVDs (4). Hence, CVD associated with thyroid dysfunction can be related to changes in the lipid profile. A cross-sectional study by Chen et al. (5) reported that 197 hypothyroid patients presented with significantly elevated levels of serum total cholesterol (TC), triglycerides (TGs), and low-density lipoprotein cholesterol (LDL-C). However, 230 hyperthyroid patients presented with lower levels of HDL-C and LDL-C. While the TG levels were positively correlated with hypothyroidism, HDL-C levels were low in both hypo- and hyperthyroid patients (6, 7). Therefore, it can be said that thyroid dysfunction in patients might affect their lipid profiles.

The treatment options for hyperthyroidism include anti-thyroid drugs (ATDs), surgical thyroid resection, symptomatic support relief, and/or radiotherapy (8). Except for several specific conditions, such as acute or subacute thyroiditis, hyperthyroid phase (i.e., thyrotoxic phase) of post-partum thyroiditis or β-hCG-mediated hyperthyroidism due to choriocarcinoma, there have been three drugs available for half a century that are recommended to treat hyperthyroidism and GD (9): ATDs such as 6-*n*-propyl-2-thiouracil (PTU), methimazole (MMI), and carbimazole (CBM). CBM is a prodrug converted to the active metabolite MMI/thiamazole. Thiamazole and MMI are the same chemical: thiamazole is the international non-proprietary name (INN) while MMI is the United States adopted name (USAN). Although PTU was introduced and approved by the United States Food and Drug Administration (US FDA) in 1947, MMI was discovered to be an even more potent and less toxic thiourea analog in 1949 (8). These drugs are thyroid peroxidase analogs that are capable of reducing the formation of T_3_ and T_4_ and blocking iodine release, thereby helping restore the plasma free T_4_ levels to a normal range (9). Several reports have shown that these drugs are associated with hepatotoxicity (10, 11).

Hyperlipidemia may cause an atherogenic effect due to an unfavorable lipid profile (12). A decrease in the level of thyroid hormones down-regulated the expression of LDL receptors-related protein 1 (LRP1) leading to decreased LDL degradation and eventually to the development of hyperlipidemia (13). In addition, previous reports have shown that reduced activities of hepatic lipase (HL), lipoprotein lipase (LPL), and cholesteryl ester transfer protein (CETP) are associated with decreased thyroid hormone levels (14). However, thyroid hormone replacement therapy was able to rescue this by up-regulating the LRP1 gene expression through the enhancement of LDL receptor mediated catabolism of LDL particles (15). Moreover, deficiency of HL has been seen to be associated with delay in atherosclerosis in an animal model (16). Additionally, down-regulation of the hepatic transcription factor, sterol regulatory element binding protein (SREBP-1c), decreases the expression of several lipogenic genes, and thus, decreases serum and hepatic TGs as well such as LDL-C and VLDL-C in DM and obesity models (17).

To date, there is no evidence about the correlation between hyperthyroidism and hyperlipidemia under several kinds of treatment in Asian and the possible underlying mechanisms. Thus, it is necessary to conduct a large-scale study to investigate whether hyperthyroidism are associated with a risk of hyperlipidemia. Although, previous meta-analyses have investigated this issue, there are major inconsistencies between these studies. Therefore, we conducted a large, nationwide cohort study using data from the Longitudinal Health Insurance Database (LHID) maintained by the National Health Research Institutes (NHRI) of Taiwan to assess the risk of hyperlipidemia associated with using different kinds of treatment among Taiwanese patients. We also investigated the possible roles of PTU and MMI in the hepatic expression of several genes involved in the production of blood lipids.

## Materials and Methods

### Data Source

The Longitudinal Health Insurance Database (LHID) was established by the National Health Research Institute (NHRI) in Taiwan. The National Health Research Institutes Database (NHIRD) contains records of patient demographics, diagnoses, examinations, drug prescriptions, and operations from all medical care settings for more than 99% of the 23 million Taiwanese. The LHID 2000 contains all of the original data for one million people who were randomly sampled from the year 2000. The NHRI confirmed that there were no significant differences in the distributions of age, sex, or health care costs between the population in the LHID and those in the NHIRD.

### Study Participants

To perform a cohort study, we selected nationwide cases of hyperthyroidism (ICD-9-CM code 242) using the data from the LHID from January 1, 2000 to December 31, 2011. We chose a cohort of patients with hyperthyroidism who had two outpatient visits or an inpatient visit for hyperthyroidism and a control cohort of normal subjects (without hyperthyroidism). The comparison cohort was randomly selected from LHID enrollees without any history of hyperthyroidism. The selection frequency consisted of 4 control patients per hyperthyroid patient and was based on age of the patients on the index date, sex, and index year. The index dates of patients with hyperthyroidism were the dates of the first definitive diagnosis. We excluded the patients who had hyperlipidemia or who lacked continuous health insurance coverage preceding the cohort entry. Additionally, we did a follow-up on the remaining patients until the earliest of hyperlipidemia outcome, National Health Insurance program withdrawal, death, or the end of study period (December 31, 2011). Patient identification numbers were encrypted for privacy protection. This study was approved by the Institutional Review Board of China Medical University Hospital (CMUH104-REC2-115-CR-4).

We followed the guidelines provided by the American Thyroid Association (ATA) (18) that, TSH, free T_4_, and T_3_ are considered to be the best markers as an initial test for patients with suspected hyperthyroidism disease. In primary hyperthyroidism, when serum TSH values are below 0.1 μIU/mL, serum free T_4_ levels are further confirmed for a diagnosis and to assess the severity of hyperthyroidism, as well as used as a benchmark for treatment decisions. If free T_4_ is increased, the diagnosis will be confirmed as clinical hyperthyroidism. Before treatment, the patient's white blood cell count and liver function were evaluated. In patients with a neutrophil count <500 mm^3^ or hepatic insufficiency, ATDs are not given, with the exception of a small number of patients who were carefully evaluated. Clinical evaluation of the patient's serum TSH and free T_4_ will be measured at 4–6 weeks into the treatment period until a euthyroid state was archived. Clinical symptoms will be tracked during all treatment periods. Since the clinical symptoms of hypothyroidism are obvious, including fatigue, increased sensitivity to cold, constipation, dry skin, heavier body weight than normal, irregular menstrual periods, slowed heart rate, and myxedema coma, these are readily recognized and the treatment will be stopped and changed accordingly.

### Outcome, Comorbidity, and Medication

The primary outcome of the study was an individual event of hyperlipidemia (ICD-9-CM code 272) is defined as increased serum fasting levels of TC (≥200 mg/dL), LDL-C (≥160 mg/dL), or TG (≥200 mg/dL), and elevations of fasting TC concentration, which may or may not be associated with the elevated TG concentration, and decreased of HDL-C (12). Blood lipid measurement is a routine medical health check up item, available for free to the public once every year and paid by the Taiwan National Health Insurance. This item is also included in the medical health check up routine and annually provided by companies or schools to the employee or students, respectively. These data also came from patients who needed to examine their blood lipid profiles data coded by the physician, if needed. The abnormal lipid profile was coded by the physician, as per the universal health insurance program; under this program the doctors are reimbursed by an administrative specialist, and a peer review system has been established to reduce the rate of false positives. Confounding factors, such as age, sex, comorbidity, and drug, were adjusted accordingly. The comorbidities and drugs were identified according to the diagnosis records in the LHID 2000 prior to the index date. Comorbidities included hypertension (ICD-9-CM code 401-405), stroke (ICD-9-CM code 430-438), diabetes mellitus (DM) (ICD-9-CM code 250), chronic obstructive pulmonary disease (COPD, ICD-9-CM code 491, 492, 496), coronary artery disease (CAD, ICD-9-CM code 410-414), alcohol-related illness (ICD-9-CM code 291, 303, 305, 571.0, 571.1, 571.2, 571.3, 790.3, A215, and V11.3), asthma (ICD-9-CM code 493), and autoimmune diseases (ICD-9-CM codes 245.2, 250.01, 340, 358, 555.9, 556.9, 579, 696.0, 696.1, 710.1, 710.2). The medication record included steroids, thiazide diuretics, and statins. We also considered the effect of hyperthyroidism drugs as a risk factor for the hyperlipidemia patients and listed the drugs, including PTU, MMI, and its prodrug, CBM.

### Chemicals and Cell Culture

All chemicals were purchased from Sigma-Aldrich (St. Louis, Missouri, USA) and were of the highest-purity grade available. The chemicals were dissolved in dimethyl sulfoxide (DMSO) at appropriate concentrations before use. The human hepatoma cell line, HepaRG™ was purchased from Thermo Fisher Scientific (Waltham, Massachusetts, USA). The frozen cells were thawed, seeded directly, and were maintained in Williams' E medium (Sigma-Aldrich, St. Louis, Missouri, USA) supplemented with 10% FetalClone™ II serum (Hyclone^TM^, GE Healthcare, Chicago, Illinois, USA), 1 × L-glutamine, 5 μg/mL human insulin, and 50 μM hydrocortisone hemisuccinate without antibiotics for 2 weeks. Next, the medium was replaced with the aforementioned medium containing 2% DMSO for 2 more weeks to induce differentiation into hepatocytes. The cells were then cultured in a humidified atmosphere of 5% CO_2_ at 37°C. The cell viability was assessed using para-nitrophenylphosphate (PNPP) as previously reported to measure their cellular acid phosphatase (ACP) activity (19).

### RNA Isolation and Quantitative Real-Time Polymerase Chain Reaction (qRT-PCR) Analysis

Total RNA was extracted from the differentiated HepaRG cells under various treatment conditions using a Direct-zol™ RNA MiniPrep kit (ZYMO Research, Irvine, CA, USA) according to the manufacturer's protocol. The quantity and purity of RNA were confirmed by ratio of absorbance at 260/280 nm. The total RNA (1 μg) was used for the synthesis of first-strand cDNA using a MultiScribe™ reverse transcriptase kit (ThermoFisher Scientific, Waltham, MA, USA). The expression of *LRP1, HL, LPL, CETP, SREBP-1c, FAS, ACLY, FAE, SCD*, and β*-actin* genes was analyzed by qRT-PCR using Luminaris Color HiGreen qPCR master mix (ThermoFisher Scientific, Waltham, MA, USA) in a StepOnePlus™ Real-Time PCR System (Applied Biosystems, ThermoFisher Scientific, Waltham, MA, USA) using standard procedures. Each pair of specific primers used for the real-time PCR analysis is listed in Table 1. The amount of target cDNA in each sample was established by determining a fractional PCR threshold cycle number (Ct value). The relative mRNA expression levels were normalized to the β*-actin* expression and the target cDNA expression was calculated using 2^−(Ct target gene−Ctβ−*actin*)^. Data are presented as fold-change compared to the control group.

**Table 1:** Sequences of PCR primers.

**Gene**	**Species**	**Forward primer (5^**′**^-3^**′**^)**	**Reverse primer (5^**′**^-3^**′**^)**
*hLRP1*	Human	ACA TAT AGC CTC CAT CCT AAT C	TTC CAA TCT CCA CGT TCA T
*hHL*	Human	TAC AGG AGT GCG GCT TCA A	TGC CAG ATC CAG TTT TCT AGC
*hLPL*	Human	CAG CAG CAA AAC CTT CAT GGT	AGT TTT GGC ACC CAA CTC TCA
*hCETP*	Human	CTG CCT GGT GGC TGG GTA TT	GGC ATC GGT CCG CAC TCT AC
*hSREBP-1c*	Human	CGC TCC TCC ATC AAT GAC AA	TGC AGA AAG CGA ATG TAG TCG AT
*hFAS*	Human	ACA TCA TCG CTG GTG GTC TG	GGA GCG AGA AGT CAA CAC GA
*hACLY*	Human	GTG TGG ACG TGG GTG ATG TG	TTG ATG TCC TCA GGA TTC AGT TTC
*hFAE*	Human	TTC CGA GTC TCC CGG AAG T	ACA GCC CAT CAG CAT CTG AGT
*hSCD*	Human	CCG ACG TGG CTT TTT CTT CT	GCG TAC TCC CCT TCT CTT TGA C
*hβ-actin*	Human	CCT GGC ACC CAG CAC AAT	GCC GAT CCA CAC GGA GTA CT

### Statistical Analysis

We used chi-square testing to determine the differences in the sociodemographic status, comorbidities, and drugs between the hyperthyroidism and comparison cohorts. Continuous variables, such as age, were showed as mean and standard deviation (SD) and analyzed using an independent *t*-test. We estimated the cumulative incidences of hyperlipidemia for both the hyperthyroidism and comparison cohorts using the Kaplan-Meier method and examined the difference between the two curves using the log-rank test. Univariate and multivariable Cox proportional regression analysis were used to measure the hazard ratio (HR) and 95% confidence interval (CI) to assess the association between hyperthyroidism and the risk of developing hyperlipidemia. The incidence density rate of hyperlipidemia (per 1,000 years) was calculated for both the hyperthyroidism and the comparison cohort. We used SAS software (version 9.4 for Windows; SAS Institute, Cary, NC, USA) for all the statistical analyses and Kaplan-Meier survival curves plot.

For *in vitro* studies, data obtained from separate measurements were reported as mean ± SD. The *p*-values for each experimental comparison were determined using ANOVA followed by Tukey's test for multiple comparisons. All *p*-values were determined relative to the vehicle control. All statistical analyses were performed using SPSS for Windows, version 20.0 (IBM SPSS, Armonk, NY, USA). A two-sided *P* < 0.05 was considered statistically significant.

## Results

### Baseline Characteristics: Demographic and Association Findings

**Figure 1:**
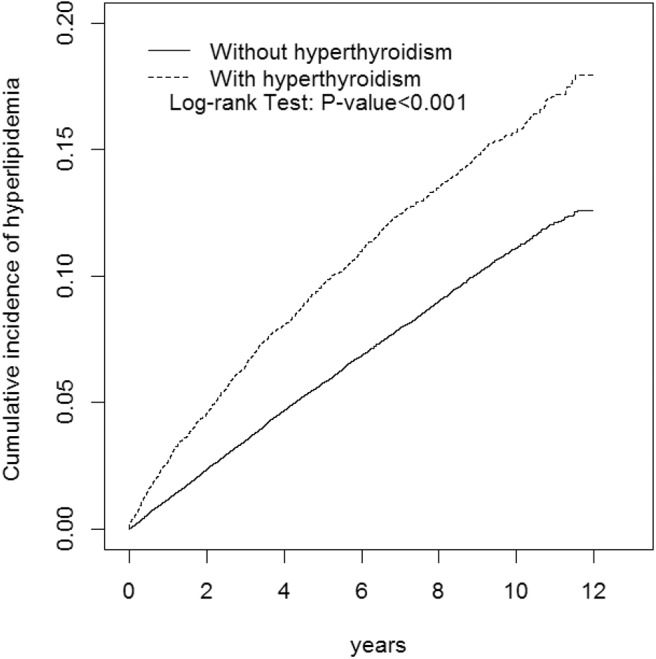
Cumulative incidence of hyperlipidemia compared between with and without hyperthyroidism using the Kaplan-Meier method. Case group mean follow-up years 5.96 (*SD* = 3.53). Control group mean follow-up years 6.08 (*SD* = 3.49).

This study involved a cohort of 13,667 patients with hyperthyroidism and a comparison cohort with four times that number (Table 2). Among the study subjects, women and individuals <34 years of age were dominant (39.3%). The mean age of the hyperthyroidism cohort and comparison cohort were 41 ± 13.8 years and 40.6 ± 14.3 years, respectively. Patients with hyperthyroidism were more likely to develop hypertension, DM, COPD, CAD, alcohol-related illness, asthma, and autoimmune diseases compared to patients without hyperthyroidism in the comparison cohort. Patients with hyperthyroidism also had higher chances of taking steroids and thiazide diuretics than those without hyperthyroidism. The mean follow-up period for hyperthyroidism and non-hyperthyroidism was 5.96 ± 3.53 and 6.08 ± 3.49 years, respectively. The Kaplan-Meier analysis result for the incidence of hyperlipidemia was significantly higher in the hyperthyroidism cohort than the comparison cohort (Figure 1).

**Table 2:** Demographic characteristics, comorbidities, and medications in patient with and without hyperthyroidism.

**Variable**	**Hyperthyroidism**		***p*-value**
	**No**	**Yes**	
	***N* = 54,668**	***N* = 13,667**	
Sex	*n* (%)	*n* (%)	0.99
Female	42,628 (78.0)	10,657 (78.0)	
Male	12,040 (22.0)	3,010 (22.0)	
Age, mean (*SD*)	40.6 (14.3)	41.0 (13.8)	0.01^#^
Stratify age			0.99
≤ 34	21,460 (39.3)	5,365 (39.3)	
35–49	20,612 (37.7)	5,153 (37.7)	
50+	12,596 (23.0)	3,149 (23.0)	
Comorbidity			
Hypertension	5,864 (10.7)	2,127 (15.6)	<0.001
Stroke	563 (1.03)	136 (1.00)	0.72
Diabetes	1,354 (2.48)	672 (4.92)	<0.001
COPD	2,083 (3.81)	849 (6.21)	<0.001
CAD	2,407 (4.40)	1,088 (7.96)	<0.001
Alcohol-related illness	975 (1.78)	296 (2.17)	<0.001
Asthma	1,941 (3.55)	703 (5.14)	<0.001
Autoimmune disease	1,178 (2.15)	608 (4.45)	<0.001
Medication			
Steroids	27,248 (49.8)	7,864 (57.5)	<0.001
Thiazide diuretics	4,665 (8.53)	1,574 (11.5)	<0.001
Statins	470 (0.86)	132 (0.97)	0.24

Chi-Square Test;

# *Two sample T-test*.

Table 3 shows the incidence rate and adjusted HR (aHR) of hyperlipidemia. The overall incidence of hyperlipidemia was higher in patients with hyperthyroidism than in patients without hyperthyroidism (18.7 and 11.8 per 1,000 person-years; aHR = 1.50). The average time until developing hyperlipidemia for the hyperthyroidism and non-hyperthyroidism groups was 3.43 years (*SD* = 2.77) and 4.00 years (*SD* = 2.81), and the number of events was 1,518 and 3,917, respectively. The patients with hyperthyroidism had higher risk of hyperlipidemia in both gender and age stratification compared to the patients without hyperthyroidism. Patients with hyperthyroidism had significantly higher risk of hyperlipidemia than the patients without hyperthyroidism without factors such as comorbidity (aHR = 1.76), steroid use (aHR = 1.51), thiazide diuretics use (aHR = 1.56), and statins use (aHR = 1.50).

**Table 3:** Comparison of incidence and hazard ratio of hyperlipidemia stratified by sex, age and comorbidity between with and without hyperthyroidism.

**Variable**	**Hyperthyroidism**							
	**No**			**Yes**				
	**Event**	**PY**	**Rate^**#**^**	**Event**	**PY**	**Rate^**#**^**	**Crude HR (95% CI)**	**Adjusted HR^**†**^ (95% CI)**
All	3917	332410	11.8	1518	81408	18.7	1.58 (1.49, 1.68)^***^	1.50 (1.41, 1.59)^***^
Sex							
Female	3002	264601	11.4	1174	64820	18.1	1.60 (1.49, 1.71)^***^	1.52 (1.42, 1.63)^***^
Male	915	67809	13.5	344	16588	20.7	1.54 (1.36, 1.74)^***^	1.41 (1.24, 1.60)^***^
Stratify age							
≤ 49	405	139586	2.90	227	34987	6.49	2.23 (1.90, 2.63)^***^	1.97 (1.67, 2.33)^***^
50-64	1707	126812	13.5	706	30669	23.0	1.71 (1.57, 1.87)^***^	1.50 (1.37, 1.64)^***^
65+	1805	66012	27.3	585	15751	37.1	1.36 (1.24, 1.49)^***^	1.27 (1.16, 1.40)^***^
Comorbidity^‡^								
No	2327	278352	8.36	790	79267	13.3	1.60 (1.47, 1.73)^***^	1.76 (1.62, 1.90)^***^
Yes	1590	54058	29.4	728	22141	32.9	1.13 (1.03, 1.23)^***^	1.18 (1.08, 1.29)^***^
Medication								
Steroids								
No	2126	183299	11.6	707	38039	18.6	1.60 (1.47, 1.74)^***^	1.51 (1.38, 1.64)^***^
Yes	1791	149111	12.0	811	43369	18.7	1.56 (1.44, 1.70)^***^	1.49 (1.37, 1.62)^***^
Thiazide diuretics								
No	3260	309426	10.5	1249	73481	17.0	1.61 (1.51, 1.72)^***^	1.56 (1.46, 1.67)^***^
Yes	657	22984	28.6	269	7927	33.9	1.19 (1.04, 1.38)^*^	1.19 (1.03, 1.37)^*^
Statins								
No	3862	330761	11.7	1493	80912	18.5	1.58(1.49, 1.68)^***^	1.50(1.41, 1.59)^***^
Yes	55	1649	33.4	25	496	50.4	1.52 (0.95, 2.44)	1.41 (0.87, 2.29)

*Rate^#^, incidence rate, per 1,000 person-years; Crude HR, crude hazard ratio*.

*Adjusted HR: multivariable analysis including age, sex, and comorbidities of hypertension, stroke, diabetes, COPD, CAD, alcohol-related illness, asthma, and autoimmune disease, and medication of steroids, thiazide diuretics, and statins*.

*Comorbidity^‡^: Patients with any one of the comorbidities hypertension, stroke, diabetes, COPD, CAD, alcohol-related illness, and asthma were classified as the comorbidity group*.

* *p < 0.05*.

*** *p < 0.001*.

Table 4 compares the incidence rates of hyperlipidemia in hyperthyroid patients with and without ATDs treatment. Hyperthyroid patients with non-use of PTU and MMI/CBM treatment showed a higher risk of hyperlipidemia (aHR = 1.58) relative to that of patients without hyperthyroidism. In addition, the hyperthyroidism patients who were only treated with PTU or MMI/CBM showed a 1.78- and 1.43-fold higher risk of hyperlipidemia than that in patients without hyperthyroidism. However, in the multivariate analyses, after controlling for age, sex, comorbidities, and medications, patients taking PTU and MMI/CBM did not have a significantly greater risk of hyperlipidemia (aHR = 1.08) compared to that of the non-hyperthyroidism cohort. Compared to patients without hyperthyroidism, hyperthyroidism patients with non-use of I^131^ treatment or surgery had a significantly higher risk of hyperlipidemia (aHR = 1.50) (Table 5). Compared to non-hyperthyroidism controls, hyperthyroidism patients with I^131^ treatment, surgery, or both also tended to have a higher risk of hyperlipidemia.

**Table 4:** Incidence, crude and adjusted hazard ratio of hyperlipidemia compared among hyperthyroidism patients with and without anti-hyperthyroidism treatment compared to non-hyperthyroidism controls.

**Variables**	***N***	**Event**	**PY**	**Rate^**#**^**	**Crude HR (95% CI)**	**Adjusted HR^**†**^ (95% CI)**
Non-hyperthyroidism controls	54,668	3917	332410	11.8	1 (Reference)	1 (Reference)
Hyperthyroidism non-use of PTU, MMI/CBM	7,259	934	45027	20.7	1.76 (1.64, 1.89)^***^	1.58 (1.47, 1.70)^***^
Only PTU	1,233	140	6677	21.0	1.77 (1.49, 2.09)^***^	1.78 (1.50, 2.11)^***^
Only MMI and CBM	3,112	300	16592	18.1	1.53 (1.36, 1.72)^***^	1.43 (1.27, 1.60)^***^
Both	2,063	144	13112	11.0	0.93 (0.79, 1.10)	1.08 (0.92, 1.28)

*Rate^#^, incidence rate, per 1,000 person-years; Crude HR, crude hazard ratio*.

*Adjusted HR: multivariable analysis including age, sex, and comorbidities of hypertension, stroke, diabetes, COPD, CAD, alcohol-related illness, asthma, and autoimmune disease, and medication of steroids, thiazide diuretics and statins*.

*** *p <0.001*.

**Table 5:** Incidence, crude and adjusted hazard ratio of hyperlipidemia compared among hyperthyroidism patients with and without I^131^, or operation treatment compared to non-hyperthyroidism controls.

**Variables**	***N***	**Event**	**PY**	**Rate^**#**^**	**Crude HR (95% CI)**	**Adjusted HR**†** (95% CI)**
Non-hyperthyroidism controls	54,668	3917	332410	11.8	1 (Reference)	1 (Reference)
Hyperthyroidism with non-use of I^131^ or operation treatment	12,409	1376	72819	18.9	1.60 (1.51, 1.70)^***^	1.50 (1.41, 1.60)^***^
With treatment						
I^131^ treatments	149	16	1051	15.2	1.30 (0.80, 2.13)	1.12 (0.69, 1.84)
Operations	1,035	114	7078	16.1	1.37 (1.14, 1.66)^***^	1.46 (1.21, 1.75)^***^
Both	74	12	459	26.1	2.21 (1.26, 3.90)^**^	2.00 (1.14, 3.53)^*^

*Rate^#^, incidence rate, per 1,000 person-years; Crude HR, crude hazard ratio*.

*Adjusted HR: multivariable analysis including age, sex, and comorbidities of hypertension, stroke, diabetes, COPD, CAD, alcohol-related illness, asthma, and autoimmune disease, and medication of steroids, thiazide diuretics and statins*.

* p < 0.05;

** *p < 0.01;*.

*** *p < 0.001*.

### Effects of PTU and MMI on Hepatic Gene Expression

**Figure 2:**
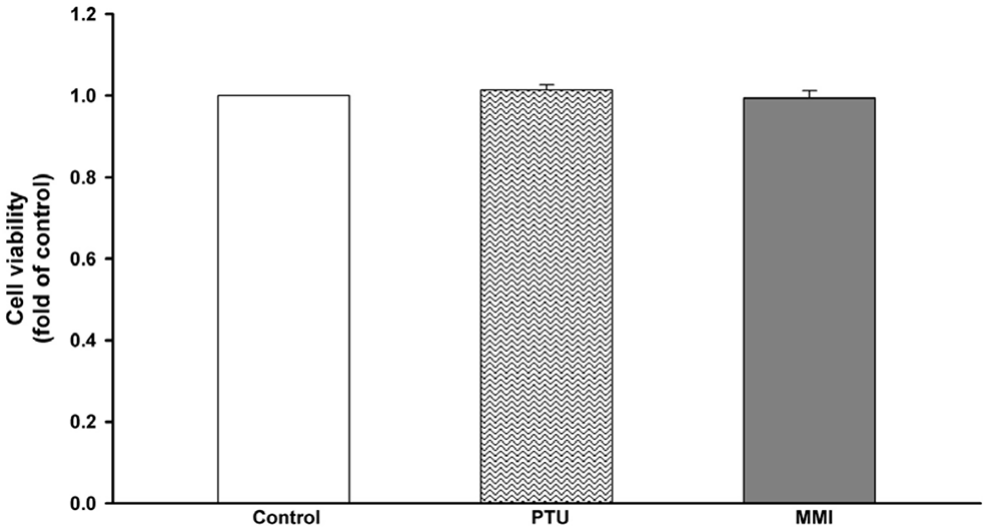
Viability of HepaRG cells following exposure to anti-thyroid drugs (ATDs), 6-*n*-propyl-2-thiouracil (PTU), and methimazole (MMI). HepaRG cells were exposed to PTU (31 μM), or MMI (3.42 μM) for 24 h. Cell viability was monitored by cellular acid phosphatase activity using PNPP as a substrate. The data shown are the mean ± SD (*n* = 3).

We used PTU and MMI to evaluate their effect on the various aspects as described below. The concentrations of drugs used were according to the maximum serum drug concentrations (20, 21). As these drugs are known to be hepatotoxic (8), we conducted a cell viability assay to evaluate their toxicity in a human hepatoma cell line, HepaRG cells. The concentrations used for PTU and MMI were 31 and 3.42 μM, respectively. HepaRG cells were treated with these ATDs for 24 h, and then subjected to ACP assay to test their cytotoxic effects. The ACP assay results showed that there was no significant difference in the cytotoxicity seen in the control and the ATDs treated cells (Figure 2).

**Figure 3:**
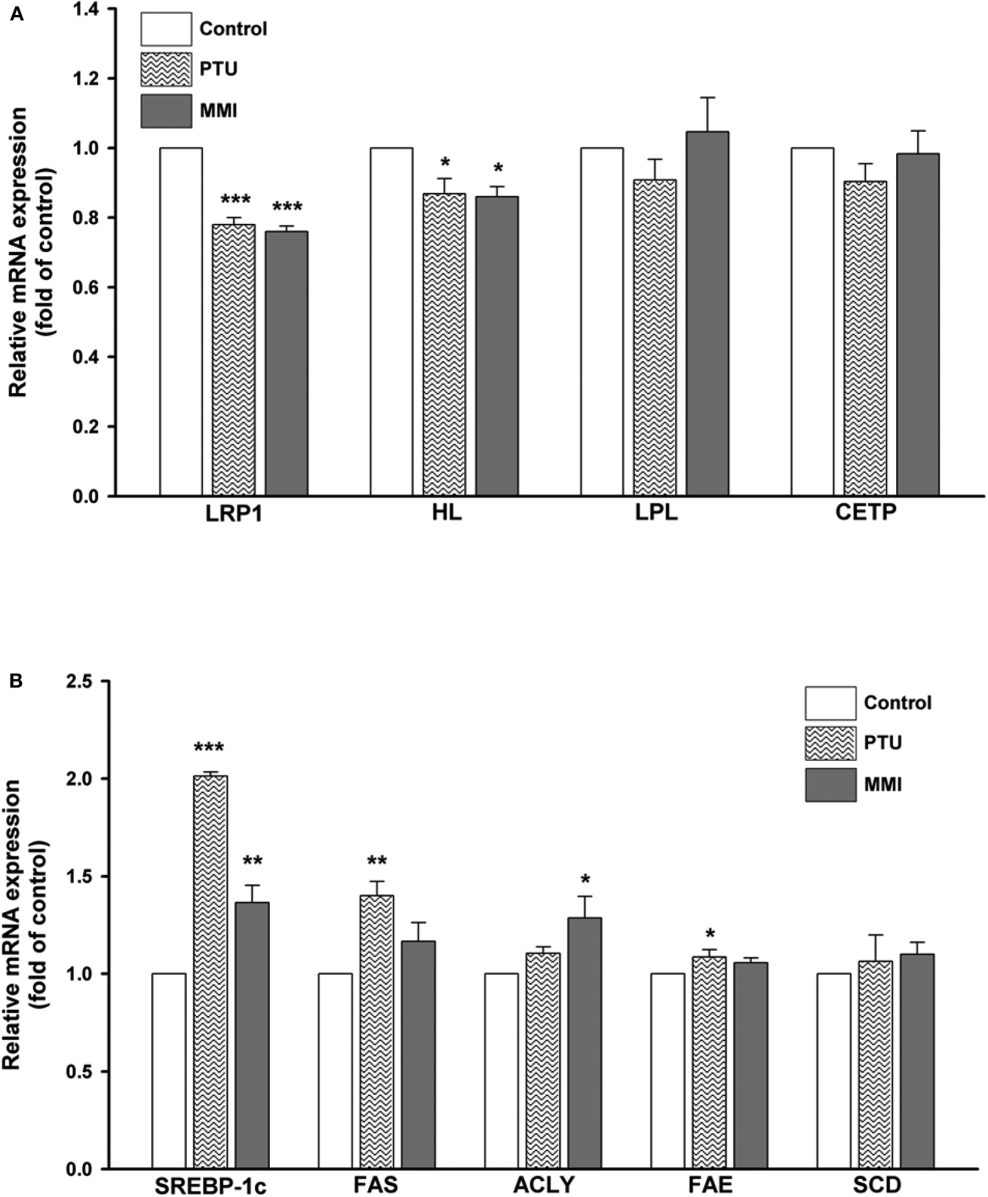
Expressions of hepatic lipid metabolism-related genes following treatment with 6-*n*-propyl-2-thiouracil (PTU), and methimazole (MMI). Differentiated HepaRG cells were treated for 24 h with PTU (31 μM) or MMI (3.42 μM). Following treatments, mRNA was extracted and the expression levels of **(A)** *LRP1, HL, LPL, CETP*, and β*-actin* **(B)** *SREBP-1c, FAS, ACLY, FAE, SCD*, and β*-actin* were analyzed by qRT-PCR. Values were normalized to the expression of β*-actin*, with the levels of DMSO-treated cells set at 1. Results are expressed as means ± SD (*n* = 3), *P < 0.05; **P < 0.01; and ***P < 0.001 compared with cells treated with DMSO.

To further obtain an overview of the effect of PTU and MMI on the expression of hepatic genes related to the circulating remnant lipoproteins, several genes encoding for *LRP1, HL, LPL, CETP*, as well as lipogenic genes such as *SREBP-1c*, fatty acid synthase (*FAS*), ATP citrate lyase (*ACLY*), fatty acid elongase (*FAE*), and stearoyl CoA desaturase (*SCD*) were measured by using real-time PCR in differentiated HepaRG cells. Differentiated HepaRG cells were treated with PTU and MMI for 24 h, used for harvesting total RNA and analysis of the expressions of the indicated genes. As shown in Figure 3A, PTU significantly reduced the gene expression of *LRP1* and *HL* to 0.78 ± 0.03- and 0.86 ± 0.08-fold of the control, respectively. MMI treatment resulted in 0.76 ± 0.02- and 0.86 ± 0.05-fold reduction in the *LRP1* and *HL* levels, respectively, compared to the control. PTU more likely reduced the gene expression of *LPL* and *CETP* than MMI, although the difference was not statistically significant. The results from the cells treated with PTU and MMI showed that the mRNA expression of *SREBP-1c* was significantly higher in treated cells than in untreated cells (2.01 ± 0.03- and 1.37 ± 0.15-fold of control, respectively). PTU was more potent than MMI in inducing the expression of *FAS* and *FAE* (1.40 ± 0.12- and 1.09 ± 0.06-fold higher than control, respectively), and MMI induced *ACLY* to 1.29 ± 0.19-fold compared to that in untreated group (Figure 3B).

## Discussion

In this study, we investigated the correlation between hyperthyroidism and hyperlipidemia in patients under treatments and our study can be summarized in the following major points: (1) this was a nationwide population-based cohort study that comprehensively surveyed the possible association between hyperthyroidism and the development of hyperlipidemia under the ATDs, I^131^, and surgery therapy; (2) after treatment with ATDs (especially PTU), the incidence of hyperlipidemia was higher in patients with hyperthyroidism than in patients without hyperthyroidism (adjusted HR = 1.78); (3) using hepatocyte-derived cell line, HepaRG, we found that PTU and MMI treatment significantly reduced the gene expression of *LRP1* and *HL* but induced expression of *SREBP-1c, FAS, ACLY*, and *FAE*; thus, they might contribute to an unfavorable blood lipid profile; (4) as a part of our data collection regarding the medical therapy for hyperthyroidism, we carried out a follow-up on the thyroid medication use and thyroid function tests and confirmed that the majority of patients with hyperthyroidism at baseline were subsequently treated; (5) to monitor the blood lipid profile and the cardiovascular prognosis in hyperthyroid patients, we performed accurate measurement of fasting lipids; (6) as this was a single cohort study with a large number of participants that was conducted for a long duration, we ensured that the outcome of our study was adjusted with most of the confounding factors.

According to a survey reported in 1991, although PTU was the first choice for ATDs in the USA, MMI was preferred as the initial treatment for hyperthyroidism in Japan and Europe (10). PTU exerts its anti-thyroid function by inhibiting the synthesis of thyroid hormones by interfering with the activity of thyroperoxidase and peripheral deiodinase (22). While this results in reduced serum T_3_ and T_4_ levels, the TSH levels are increased to prevent the induction of hypothyroidism (23). Previous reports have compared the adverse effects of PTU (at a dose of 300 mg/d) and MMI (at doses of 15 and 30 mg/d) and they concluded that the incidence of hepatotoxicity was higher in the PTU group (24). Persistent exposure to PTU in neonatal rats leads to the induction of oxidative stress in the livers of these rats during adulthood (23). Moreover, US FDA has issued a “black box alert” regarding PTU due to its hepatotoxicity (24). However, PTU is recommended for patients who are allergic to MMI and for pregnant women as MMI has been shown to be more teratogenic than PTU (9, 24). In addition, PTU treatment, at a dose of 400 mg/kg body weight along with piperine, was found to significantly increase the serum levels of TC, TG, LDL-C, and VLDL-C and the induction of HDL-C (all *p* < 0.01) (25). Moreover, studies conducted using chicks have shown that PTU induces hypothyroidism along with hyperlipidemia followed by development of severe toxic effects after clofibate administration (26). Traditionally, MMI has been considered to be more teratogenic than PTU. MMI at a dose of 30 mg/d stabilized the thyroid hormone levels more rapidly than PTU at a dose of 300 mg/d (6.7 ± 4.6 week vs. 16.8 ± 13.7 week, respectively) (27, 28) and hence, MMI is considered to be more effective. A randomized control trial showed that in patients with severe hyperthyroidism, treatment with 30 mg/d of MMI is more effective in achieving normal thyroid hormone status than 300 mg/d of PTU or 15 mg/d of MMI (10). PTU induced mild liver damage which was four times higher than that induced by MMI treatment (30 mg/d) or even severe hepatic toxicity (10, 23, 29). Hence, it was suggested that MMI (15 mg/d) is more suitable for treating patients with mild to moderate GD due to lesser chances of adverse effects. From our results, we showed that patients treated with PTU showed higher hazard ratio of developing hyperlipidemia than those treated with MMI/CBM (1.78 vs. 1.43).

The association between decreased thyroid hormone levels and hyperlipidemia has been consistently reported as many researchers have established that dyslipidemia is associated with thyroid dysfunction (4, 30, 31). In the present cohort-based study, we found that hyperthyroidism patients without hyperlipidemia undergoing standard ATDs treatment showed significantly higher risk of developing hyperlipidemia after follow-up. Several possibilities were proposed that included decreased thyroid hormones during therapy period, attenuated activity of lipoprotein lipase (LPL) leading to reduced clearance of TG-rich lipoproteins (VLDL, and chylomicron), and thus, increasing the TG levels in the serum of the patients (32). Moreover, T_3_ can up-regulate LDL receptors by directly binding to the thyroid hormone responsive elements (TREs) (33). Due to the reduced expression of LDL receptors, there is a decrease in the uptake of LDL-C from the circulation leading to increased serum levels of LDL-C and consequently, leading to increased risk of CVD. Moreover, thyroid hormones also increase the activity of CETP and trigger the exchange of HDL to VLDL (34). In addition, T_3_ was involved in protecting LDL particles from oxidation (35). Therefore, due to low T_3_ levels, there are circulating lipoprotein remnants that are taken up by macrophages found in the arterial walls to produce foam cells resulting in the formation of atherosclerotic plaques.

The atherogenic effects of decreased thyroid hormones on lipid metabolism could result, in part, from the reduced clearance of remnant lipoproteins. Thyroid hormones can influence HDL metabolism by increasing CETP activity, which exchanges cholesteryl esters from HDL2 to the VLDL and from TGs to HDLs (35). A rare mutation in CETP leading to its reduced function has been linked with accelerated atherosclerosis (36). In addition, thyroid hormones can stimulate LPLs and the HLs that can catabolize the TG-rich lipoproteins and hydrolyze HDL2 to HDL3, respectively, contributing to the conversion of IDL to LDL and subsequently, LDL to sdLDL (37). Hence, LPL deficiency is responsible for hypertriglyceridemia. In this study, we investigated the effect of PTU and MMI treatment on the expression of gene expression of hepatic *LRP1* (a receptor for remnant lipoproteins), *HL, LPL*, and *CETP*. We found significant decrease in the expression of *LRP1* and *HL* following treatment with PTU and MMI. Further, the decrease in the expression of *LPL* and *CETP* genes were higher in the PTU group than the MMI group, although without any statistically significant difference.

In a study carried out by Moon et al. (38), hypothyroidism was induced in the C57BL/6 mice that were fed with a low-iodine diet supplemented with 0.15% PTU as opposed to the control mice that were fed with a normal diet. The expression of hepatic LRP1 protein was found to be much lower in the PTU group compared with the control mice. However, upon treatment with T_3_, LRP1 protein expression was upregulated in PTU-treated mice. LRP1, a member of the LDL receptor gene family, is expressed on the surface of the hepatocytes and acts as a multifunctional signaling receptor by internalizing various ligands (13). LRP1 binds to and internalizes TG-rich lipoproteins containing ApoE, such as chylomicron remnants and VLDL remnants (13). Conversely, LDL receptors recognize LDL (containing apolipoprotein B) and bind to lipidated ApoE. Although LRP1 has no role in the hepatic uptake and clearance of circulating LDL, it is mainly responsible for clearing remnant lipoproteins (accounting for ~80% of the LDL receptor–mediated clearance of chylomicron remnants) (38, 39). These hepatic receptors that are responsible for the clearance of circulating chylomicrons and VLDL/LDL remnants could be affected by alterations in thyroid hormone levels. Further, they found that in HepG2 cells cultured in the presence of charcoal-stripped fetal bovine serum, the expression of *LRP1* was reduced, which mimics hypothyroidism *in vitro*, and was recovered by T_3_ treatment in a dose dependent manner up to 2 nmol/L T_3_ (38). However, they did not investigate the effect of PTU or MMI on the expression of *LRP1* in hepatic cells. Our data demonstrated that when PTU and MMI reach the plasma concentrations, the *LRP1* expression is decreased in the hepatic cells. These results suggest that in hyperthyroid patients undergoing ATDs treatment, along with the decrease in thyroid hormones, the drugs have inhibitory effects on the genes expression of *LRP1* and *HL* that may lead to reduced clearance of circulating remnant lipoproteins.

PTU and MMI were also found to elevate *de novo* hepatic lipogenesis. Patients suffering from fatty liver disease weighed more and had a greater incidence of hypertriglyceridemia (44 vs. 27%), hypercholesterolemia (54 vs. 44%), and decreased HDL-C (15 vs. 8%) (40). They also had significantly greater serum levels of TG and non-significantly greater levels of total cholesterol. Serum TGs concentration >130 mg/dL is a good predictor of fatty liver in Taiwanese, and it is positively correlated with the degree of severity of fatty liver (41). Therefore, it is possible that ATDs cause hyperlipidemia by increasing the expression of hepatic lipogenic-related genes.

To our knowledge, this is the first population-based nationwide cohort study to evaluate the potential role of ATDs in the development of hyperlipidemia in hyperthyroidism patients. However, our study contains several limitations. First, the patient information concerning life behavior, smoking status, alcohol consumption, environmental exposure, body mass index, and family history of hyperlipidemia, were unavailable in the LHIRD. These factors may act as confounding factors as they are linked with the possibility of development of hyperlipidemia. Second, LHIRD claims data are used mainly for administrative billing purposes and hence, the additional information was anonymous. Therefore, we were no able to contact the patient directly for any additional information. Third, due to the lack of information, we could not evaluate the dietary effects in this study as dietary intake types may contribute to the control of patients' lipid profile. Fourth, the availability of specific laboratory data in LHIRD was limited, and therefore we could not make underlying interferences between variables, such as thyroid hormones, and TSH. Since the data were based on a publicly available database from Taiwan's NHIRD without corroborating laboratory data, which is a barrier for conducting certain types of pharmacoepidemiology studies. In particular, determining disease severity is a challenge (for example, the lipid profile in hyperlipidemia). Fifth, the LHIRD lacks complete information regarding liver disease in the patients based on Child-Pugh classification, which might highly correlate with the development of hyperlipidemia.

## Conclusion

In summary, our results provide evidence that hyperthyroid patients undergoing treatments have chances of developing hyperlipidemia that might result in long-term consequences, such as CVD. Hence, based on these results, recommendations and guidelines should be provided for the clinical management of hyperthyroid patients to effectively mitigate the possibility of hyperlipidemia-related CVD in these patients. Treatment of hyperthyroid patients with PTU and MMI reduces their thyroid hormone status and the hepatic expression of genes involved in the clearance of lipoproteins as well as increases the levels of lipogenic-related genes, thus leading to abnormal blood lipid profiles.

## Data Availability Statement

The datasets generated for this study are available on request to the corresponding authors.

## Ethics Statement

The NHIRD encrypts patient personal information to protect privacy and provides researchers with anonymous identification numbers associated with relevant claims information, including sex, date of birth, medical services received, and prescriptions. Therefore, patient consent is not required to access the NHIRD. This study was approved to fulfill the condition for exemption by the Institutional Review Board (IRB) of China Medical University (CMUH104-REC2-115-CR-4). The IRB also specifically waived the consent requirement.

## Author Contributions

T-YW, C-HW, NT, and Y-PL: conceptualization and supervision. T-YW, C-LL, F-YC, and Y-PL: methodology. C-LL, H-YC, and Y-PL: software. T-YW and NT: validation. C-LL and F-YC: formal analysis. T-YW and Y-PL: investigation. C-LL and Y-PL: resources and data curation. Y-PL: project administration. C-HW and Y-PL: funding acquisition. All authors: writing—original draft preparation, writing—review and editing, and visualization.

## Conflict of Interest

The authors declare that the research was conducted in the absence of any commercial or financial relationships that could be construed as a potential conflict of interest.

## Abbreviations

ACLY: ATP citrate lyase
ACP: acid phosphatase
ATDs: anti-thyroid drugs
CAD: coronary artery disease
CBM: carbimazole
CETP: cholesteryl ester transfer protein
CI: confidence interval
COPD: chronic obstructive pulmonary disease
CVD: cardiovascular disease
DMSO: dimethyl sulfoxide
FAE: fatty acid elongase
FAS: fatty acid synthase
GD: Graves' disease
HDL-C: high density lipoprotein cholesterol
HL: hepatic lipase
HR: hazard ratio
LDL-C: low density lipoprotein cholesterol
LHID: Longitudinal Health Insurance Database
LPL: lipoprotein lipase
LRP1: LDL receptors-related protein 1
MMI: methimazole
NHRI: National Health Research Institutes
NHRID: National Health Research Institutes Database
PNPP: para-nitrophenylphosphate
PTU: 6-*n*-propyl-2-thiouracil
SCD: stearoyl CoA desaturase
SREBP-1c: sterol regulatory element binding protein 1c
T_3_: triiodothyronine
T_4_: thyroxine
TC: total cholesterol
TGs: triglycerides
TSH: thyroid stimulating hormone
VLDL-C: very low density lipoprotein lipoprotein.
